# Predicting Tracheostomy Need on Admission to the Intensive Care Unit—A Multicenter Machine Learning Analysis

**DOI:** 10.1002/ohn.919

**Published:** 2024-07-30

**Authors:** Matthew Nguyen, Ameen Amanian, Meihan Wei, Eitan Prisman, Pedro Alejandro Mendez‐Tellez

**Affiliations:** ^1^ UVA School of Medicine Charlottesville Virginia USA; ^2^ Department of Surgery, Division of Otolaryngology–Head & Neck Surgery University of British Columbia Vancouver Canada; ^3^ Department of Biomedical Engineering–Whiting School of Engineering Johns Hopkins University Baltimore USA; ^4^ Department of Anesthesiology and Critical Care Medicine Johns Hopkins University School of Medicine Baltimore USA

**Keywords:** Artificial Intelligence, machine learning, mechanical ventilation, outcome prediction, tracheostomy

## Abstract

**Objective:**

It is difficult to predict which mechanically ventilated patients will ultimately require a tracheostomy which further predisposes them to unnecessary spontaneous breathing trials, additional time on the ventilator, increased costs, and further ventilation‐related complications such as subglottic stenosis. In this study, we aimed to develop a machine learning tool to predict which patients need a tracheostomy at the onset of admission to the intensive care unit (ICU).

**Study Design:**

Retrospective Cohort Study.

**Setting:**

Multicenter Study of 335 Intensive Care Units between 2014 and 2015.

**Methods:**

The eICU Collaborative Research Database (eICU‐CRD) was utilized to obtain the patient cohort. Inclusion criteria included: (1) Age >18 years and (2) ICU admission requiring mechanical ventilation. The primary outcome of interest included tracheostomy assessed via a binary classification model. Models included logistic regression (LR), random forest (RF), and Extreme Gradient Boosting (XGBoost).

**Results:**

Of 38,508 invasively mechanically ventilated patients, 1605 patients underwent a tracheostomy. The XGBoost, RF, and LR models had fair performances at an AUROC 0.794, 0.780, and 0.775 respectively. Limiting the XGBoost model to 20 features out of 331, a minimal reduction in performance was observed with an AUROC of 0.778. Using Shapley Additive Explanations, the top features were an admission diagnosis of pneumonia or sepsis and comorbidity of chronic respiratory failure.

**Conclusions:**

Our machine learning model accurately predicts the probability that a patient will eventually require a tracheostomy upon ICU admission, and upon prospective validation, we have the potential to institute earlier interventions and reduce the complications of prolonged ventilation.

Across the United States, ~900,000 patients experienced mechanical ventilation,^1^ of which approximately 34% underwent a tracheostomy secondary to prolonged mechanical ventilation (PMV).^2^ Approximately 60,000 tracheostomy tubes on averaged are placed across the United States each year.^3^ This is often for several general indications such as prolonged intubation, allowance of weaning from the ventilator, upper airway obstruction, and airway protection in neurologic disease or traumatic brain injury.^4^, ^5^ Specifically, prolonged intubation may result in a myriad of airway‐related complications such as laryngotracheal stenosis, vocal fold immobility, vocal fold granulomas, dysphagia, dysphonia, tracheomalacia and tracheoesophageal fistulas amongst others.^1^, ^6^, ^7^, ^8^, ^9^ However, tracheostomies also pose several possible complications including pneumothorax, bleeding, subglottic stenosis, tracheoesophageal fistula, vocal cord dysfunction, stomal granulation, persistent tracheal fistula, and scarring.^10^ Nevertheless, there is significant variability in selecting when and who should receive a tracheostomy in the ICU.^11^, ^12^ Kishihara et al in a network meta‐analysis of 8 randomized control trials evaluating the tracheostomy timing after mechanical ventilation on patient prognosis, observed a possible reduction in short‐term mortality in tracheostomies performed <4 days after intubation compared to those performed >13 days after intubation.^13^ However, they noticed no significant difference in adverse events when comparing the same 2 time ranges for tracheostomy. Noemie et al in a meta‐analysis of 9 trials concluded no effect on overall mortality, ICU length of stay, duration of MV, or rates of ventilator‐acquired pneumonia in patients who received a tracheostomy <10 days versus ≥10 days after intubation.^14^

In the setting of PMV, patients who received early tracheostomies experienced lower in‐hospital mortality, greater weaning rates, and shorter ICU length of stay.^15^, ^16^ Furthermore, tracheostomy offers several advantages over intubation: reduced need for sedation, facilitation of weaning, and improved patient communication and swallowing.^7^, ^13^, ^17^, ^18^, ^19^ Despite these benefits and the common practice of transitioning patients to a tracheostomy tube between 1 and 3 weeks after intubation, there is no clear guidance on when to perform this transition.^20^, ^21^, ^22^ Several factors have been associated with undergoing a tracheostomy in the ICU. Patients who developed nosocomial pneumonia, received administration of aerosol treatments, experienced an aspiration event, and underwent unsuccessful extubation requiring reintubation were significantly related to the likelihood of requiring a tracheostomy.^23^ The validity of these associations in predicting tracheostomy need however yet to be proven. Given the risks, morbidities, and increasing mortality associated with prolonged ventilation, a tool that incorporates patient‐specific demographic, comorbidities, physiologic and clinical features, and ventilation variables that could predict an individualized need for tracheostomy would be valuable for clinical practice and might allow appropriate timing of early tracheostomies.

As computational power and big data grow in popularity, new methods of efficiently and effectively exploring and studying massive amounts of clinical data have been used.^24^ One such class of methods has taken the clinical research world by storm, machine learning (ML), for its possible role in producing personalized predictions based on large sums of data. More specifically, it may have a role in better‐identifying patients who need a tracheostomy upon admission to the ICU. Therefore, the objective of this study was to use a large public dataset to develop and validate an ML model for predicting the likeliness of needing a tracheostomy for patients in the ICU upon admission. We hope that our model would help reduce the duration of MV for ICU patients, improve patient outcomes, and enhance the collaborative relationship between the tracheostomy interventionalist and the ICU.

## Methods

The study's conduct and results are reported per the transparent reporting of a multivariable prediction model for individual prognosis or diagnosis (TRIPOD) statement in Table 1.^25^

**Table 1:** TRIPOD Checklist: Prediction Model Development

Section/topic	Item	Checklist Item	Page
Title and abstract			
Title	1	Identify the study as developing and/or validating a multivariable prediction model, the target population, and the outcome to be predicted.	1
Abstract	2	Provide a summary of objectives, study design, setting, participants, sample size, predictors, outcome, statistical analysis, results, and conclusions.	3
Introduction			
Background and objectives	3a	Explain the medical context (including whether diagnostic or prognostic) and rationale for developing or validating the multivariable prediction model, including references to existing models.	4
	3b	Specify the objectives, including whether the study describes the development or validation of the model or both.	5
Methods			
Source of data	4a	Describe the study design or source of data (e.g., randomized trial, cohort, or registry data), separately for the development and validation data sets, if applicable.	6
	4b	Specify the key study dates, including start of accrual; end of accrual; and, if applicable, end of follow‐up.	6
Participants	5a	Specify key elements of the study setting (e.g., primary care, secondary care, general population) including number and location of centres.	6
	5b	Describe eligibility criteria for participants.	6
	5c	Give details of treatments received, if relevant.	6
Outcome	6a	Clearly define the outcome that is predicted by the prediction model, including how and when assessed.	7
	6b	Report any actions to blind assessment of the outcome to be predicted.	7
Predictors	7a	Clearly define all predictors used in developing or validating the multivariable prediction model, including how and when they were measured.	7
	7b	Report any actions to blind assessment of predictors for the outcome and other predictors.	7
Sample size	8	Explain how the study size was arrived at.	6
Missing data	9	Describe how missing data were handled (e.g., complete‐case analysis, single imputation, multiple imputation) with details of any imputation method.	6
Statistical analysis methods	10a	Describe how predictors were handled in the analyses.	6
	10b	Specify type of model, all model‐building procedures (including any predictor selection), and method for internal validation.	8
	10d	Specify all measures used to assess model performance and, if relevant, to compare multiple models.	8
Risk groups	11	Provide details on how risk groups were created, if done.	6
Results			
Participants	13a	Describe the flow of participants through the study, including the number of participants with and without the outcome and, if applicable, a summary of the follow‐up time. A diagram may be helpful.	9
	13b	Describe the characteristics of the participants (basic demographics, clinical features, available predictors), including the number of participants with missing data for predictors and outcome.	24
Model development	14a	Specify the number of participants and outcome events in each analysis.	24
	14b	If done, report the unadjusted association between each candidate predictor and outcome.	23
Model specification	15a	Present the full prediction model to allow predictions for individuals (i.e., all regression coefficients, and model intercept or baseline survival at a given time point).	29
	15b	Explain how to the use the prediction model.	9
Model performance	16	Report performance measures (with CIs) for the prediction model.	9
Discussion			
Limitations	18	Discuss any limitations of the study (such as nonrepresentative sample, few events per predictor, missing data).	14
Interpretation	19b	Give an overall interpretation of the results, considering objectives, limitations, and results from similar studies, and other relevant evidence.	13
Implications	20	Discuss the potential clinical use of the model and implications for future research.	13
Other information			
Supplementary information	21	Provide information about the availability of supplementary resources, such as study protocol, Web calculator, and data sets.	NA
Funding	22	Give the source of funding and the role of the funders for the present study.	2

### Data Source

The data for this study was extracted from the eICU Collaborative Research Database (eICU‐CRD), a publicly available and fully deidentified clinical database for over 139,367 patients admitted to 1 of 335 units at 208 hospitals between 2014 and 2015.^26^ All patients in the eICU‐CRD dataset were eligible for inclusion. Patients who were (1) Age 18 years or older and (2) had an ICU admission requiring mechanical ventilation were included. Only data of the first ICU admission of the first hospitalization were used for each patient. Outliers were defined as 3.5 standard deviations outside of the mean in each feature and eliminated.^27^ Incomplete patient data were imputed using multiple imputations by chained equations (MICE).^28^ This method models the relationships between variables of the original data. It imputes the missing values with estimates that preserve inter‐variable relationships and the original data's distributions. The study was exempt from institutional review board approval due to the retrospective design, lack of direct patient intervention, and the security schema, for which the re‐identification risk was certified as meeting safe harbor standards by an independent privacy expert (Privacert) (Health Insurance Portability and Accountability Act Certification no. 1031219‐2).

### Study Population

**Figure 1:**
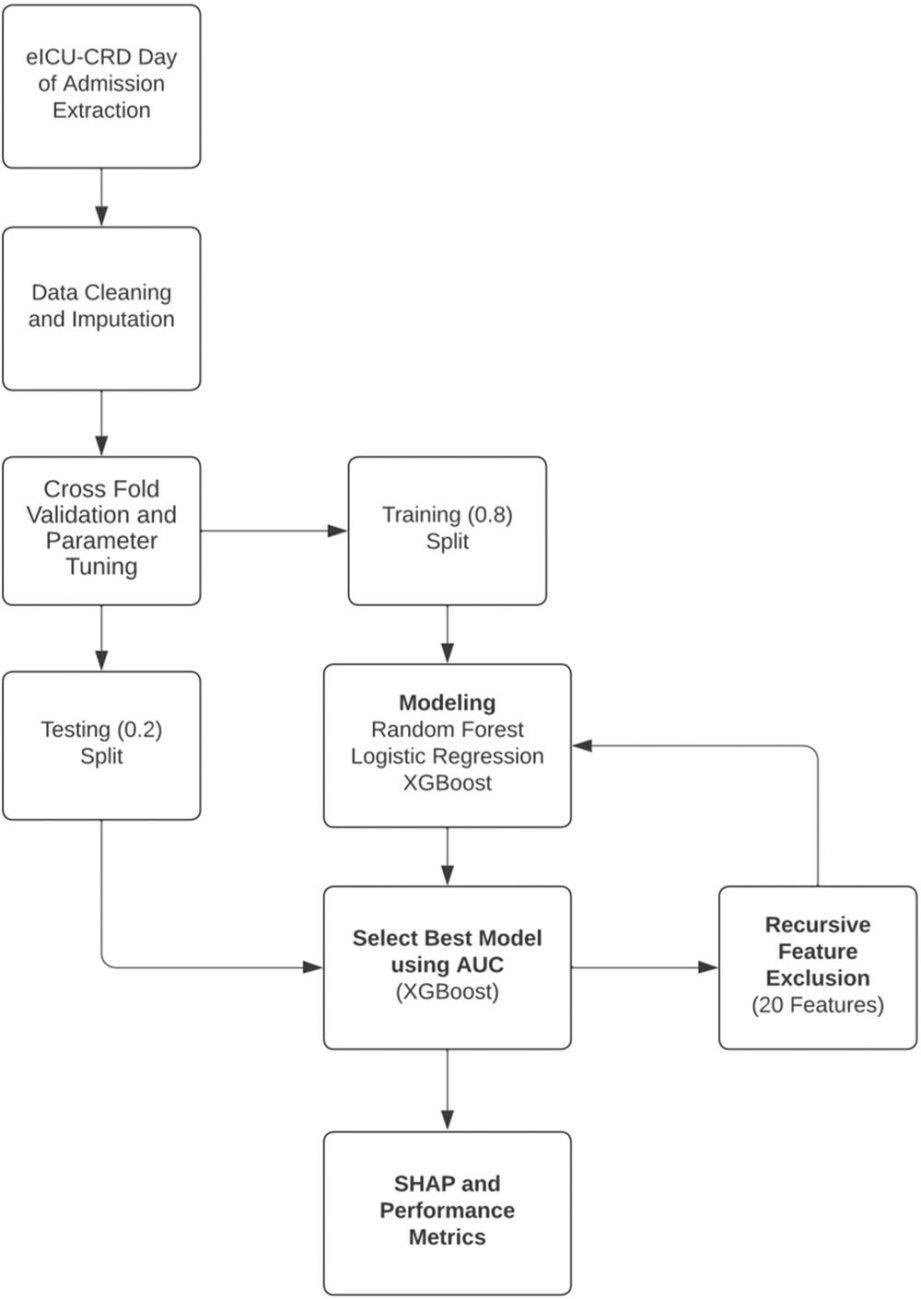
Overall project workflow.

Figure 1 demonstrates the workflow of the clinical study from data extraction to prediction model development and performance assessment. The selection of variables extracted was based on a literature review, clinical judgment of factors that would potentially contribute to the likelihood of tracheostomy, and overall dataset exploration. Data were extracted from the database using structured query language (SQL) and included the following demographic data (using the first 24 hours of admission data): age, sex, race, height, weight, comorbidities, and disease severity scores (Acute Physiology Score [APS] III, Logistic Organ Dysfunction System [LODS], Sequential Organ Failure Assessment [SOFA], Simplified Acute Physiology Score [SAPS] III, APACHE).^29^, ^30^, ^31^, ^32^ Laboratory measurements (eg, arterial blood gas, albumin), were captured as the lowest and the highest values in the first day of ventilation. All ventilation variables were extracted as the highest and lowest values during the first 24 hours after admission (eg, ventilation mode, positive end‐expiratory pressure [PEEP] level, tidal volume [*V*_T_]). The full list of extracted variables can be seen in Table 2. For variables that included multiple measurements throughout the day (eg, PEEP), we calculated the minimum, mean, and maximum pertaining to the variable. The primary prediction outcome was a tracheostomy, defined as the binary variable for the machine learning model.

**Table 2:** Features Used for Predicting “Tracheostomy Need”

Patient demographics	Comorbidities	Disease severity scores	Lab results	Ventilation settings
Age Sex Caucasian Other/Unknown Native American African American Asian Hispanic	AIDS Angina Atrial Fibrillation Coronary Artery Bypass Graft Congestive Heart Failure COPD Diabetes Deep Venous Thrombosis HIV Lymphoma Myocardial Infarction Thyroid Dysfunction Pulmonary Embolism Renal Failure (on dialysis) Renal Failure Chronic Respiratory Failure Transplant Seizures Stroke Transient Ischemic Attack Upper Gastrointestinal Bleed Ventricular arrhythmia Steroids Autoimmune Chemotherapy Dementia Home Oxygen	SOFA qSOFA SAPSII OASIS LODS APACHEIV APSIII	Anion Gap Albumin Bicarbonate Total Bilirubin Creatinine Chloride Glucose Hematocrit Hemoglobin Lactate Platelets Potassium PTT INR PT Sodium BUN WBC Temperature SaO2 Heartrate Respiration CVP Systolic BP Diastolic BP	Machine Tidal Volume Patient Tidal Volume Total Respiratory Rate PIP PEEP paO_2_ pCO_2_ FiO_2_

Admission diagnosis			
Acute Respiratory Failure Pneumonia Sepsis Acute Renal Failure Hypertension Hyperglycemia Atrial Fibrillation Congestive Heart Failure Hypoxemia Cardiac Arrest Septic Shock Stroke Change in Mental Status Encephalopathy Anemia Intracranial Injury Seizures COPD	Pleural Effusion Hypokalemia Acute Respiratory Distress Syndrome Metabolic Acidosis Acute Coronary Syndrome Bone fracture(s) Leukocytosis Nutritional Deficiency Thrombocytopenia Hepatic Dysfunction Hyperlipidemia Signs and Symptoms of Sepsis (SIRS) Hypothyroidism Hypovolemia Coagulopathy Hypomagnesemia	Fever Hematological Effect of Infection Lower Urinary Tract Infection Hyponatremia Agitation Sinus Tachycardia Atelectasis/Collapse Coronary Artery Disease Upper GI bleeding Acute COPD Exacerbation Hypernatremia ESRD (end‐stage renal disease) Obesity	Abdominal Pain/tenderness Bacteremia Depression Chronic Respiratory Failure Cardiogenic shock Hyperkalemia Hypocalcemia Hypercarbia Drug Withdrawal Syndrome Hypervolemia Acute Pulmonary Edema Urinary Tract Infection Delirium Lung trauma Pneumothorax Deep Venous Thrombosis

Abbreviations: AIDS, Acquired Immune Deficiency Syndrome; APACHEIV, Acute Physiology And Chronic Health Evaluation IV; APSIII, Acute Physiology Score III; BUN, blood urea nitrogen; COPD, Chronic Obstructive Pulmonary Disease; CVP, Central Venous Pressure; FiO_2_, fraction of inspired oxygen; HIV, human immunodeficiency virus; INR, international normalized ratio; LODS, Logistic Organ Dysfunction System; OASIS, Oxford Acute Severity of Illness Score; paO_2_, partial pressure of arterial oxygen; pCO_2_, partial pressure of carbon dioxide; PEEP, positive end‐expiratory pressure; PIP, peak inspiratory pressure; PT, prothrombin time; PTT, partial thromboplastin time; qSOFA, quick sequential organ failure assessment; SaO_2_, arterial oxygen saturation; SAPSII, Simplified Acute Physiology Score II; SOFA, Sequential Organ Failure Assessment; WBC, white blood cell count.

### Tracheostomy Identification

To first identify all ICU patients who underwent invasive mechanical ventilation, an SQL script provided by the Massachusetts Institute of Technology (MIT) was used to extract the type of oxygen therapy patients received while admitted.^26^ Then, patients were separated based on whether they had received a tracheostomy after they were placed on the mechanical ventilator as reflected in the eICU‐CRD respiratory charting and treatment tables.

### Model Development

We used patient demographics, comorbidities, day of admission (DOA) clinical variables, and traditional disease severity scoring systems such as SOFA and APS‐III to create the dataset (Table 2). The dataset was randomly divided into a training and testing set with a ratio of 80% and 20% respectively. The training set was used to develop the ML models, while the testing set was used to evaluate their performance. To optimize the hyperparameters that dictate how the models train on the dataset, the data was split into 5 groups, and the models were iteratively trained and optimized within each of the 5 portions of the training data. The best hyperparameters were taken as an average of the model's performance across the 5 groups in a 5‐fold nested cross‐validation (Table 3). Recursive feature elimination (RFE) was utilized to select the top 20 predictive features within the dataset. RFE iteratively reduces the number of features to train the model while minimizing loss in AUC‐ROC performance, resulting in 20 remaining features that most contribute to the model's ability to predict tracheostomy need. Following data pruning via RFE, the top 20 features were selected to re‐train the model and its performance was then compared to the model with all included features before RFE. The model features were then analyzed using several supervised ML algorithms, including random forest (RF), logistic regression (LR), and eXtreme Gradient Boosting (XGBoost). The models’ performance was evaluated by several metrics (Table 2) such as the precision‐recall curve (PR), the area under the receiver operator characteristic curve (AUC‐ROC), and the F1‐score. Finally, to gain insights into the most relevant predictors of tracheostomy need and to determine the relative contributions of different variables to the overall tracheostomy need, a feature importance analysis was conducted using the Shapley Additive Explanations (SHAP) framework.

**Table 3:** Hyperparameters Used for Each Classification Algorithm

Hyperparameter	XGBoost	Hyperparameter	Random forest	Hyperparameter	Logistic regression
objective'	'binary	'ccp_alpha'	0.0,	C'	2.7990334400837247,
'use_label_encoder'	None	'class_weight'	None,	'class_weight'	None,
'base_score'	None	'criterion'	'gini',	'dual'	False,
'booster'	None	'max_depth'	21,	'fit_intercept'	True,
'callbacks'	None	'max_features'	'sqrt',	'intercept_scaling'	1,
'colsample_bylevel'	None	'max_leaf_nodes'	None,	'l1_ratio'	None,
'colsample_bynode'	None	'max_samples'	None,	'max_iter'	201,
'colsample_bytree'	None	'min_impurity_decrease'	0.0,	'multi_class'	'auto',
'early_stopping_rounds'	None	'min_samples_leaf'	81,	'n_jobs'	None,
'enable_categorical'	False	'min_samples_split'	43,	'penalty'	'l1',
'eval_metric'	None	'min_weight_fraction_leaf'	0.0,	'random_state'	None,
'feature_types'	None	'n_estimators'	476,	'solver'	'saga',
'gamma'	5	'n_jobs'	None,	'tol'	0.01,
'gpu_id'	None	'oob_score'	False,	'verbose'	0,
'grow_policy'	None	'random_state'	None,	'warm_start'	False
'importance_type'	None	'verbose'	0,		
'interaction_constraints'	None	'warm_start'	False},		
'learning_rate'	None				
'max_bin'	None				
'max_cat_threshold'	None				
'max_cat_to_onehot'	None				
'max_delta_step'	None				
'max_depth'	848				
'max_leaves'	None				
'min_child_weight'	None				
'missing'	Nan				
'monotone_constraints'	None				
'n_estimators'	970				
'n_jobs'	None				
'num_parallel_tree'	None				
'predictor'	None				
'random_state'	None				
'reg_alpha'	None				
'reg_lambda'	None				
'sampling_method'	None				
'scale_pos_weight'	None				

### Statistical Analysis

**Figure 2:**
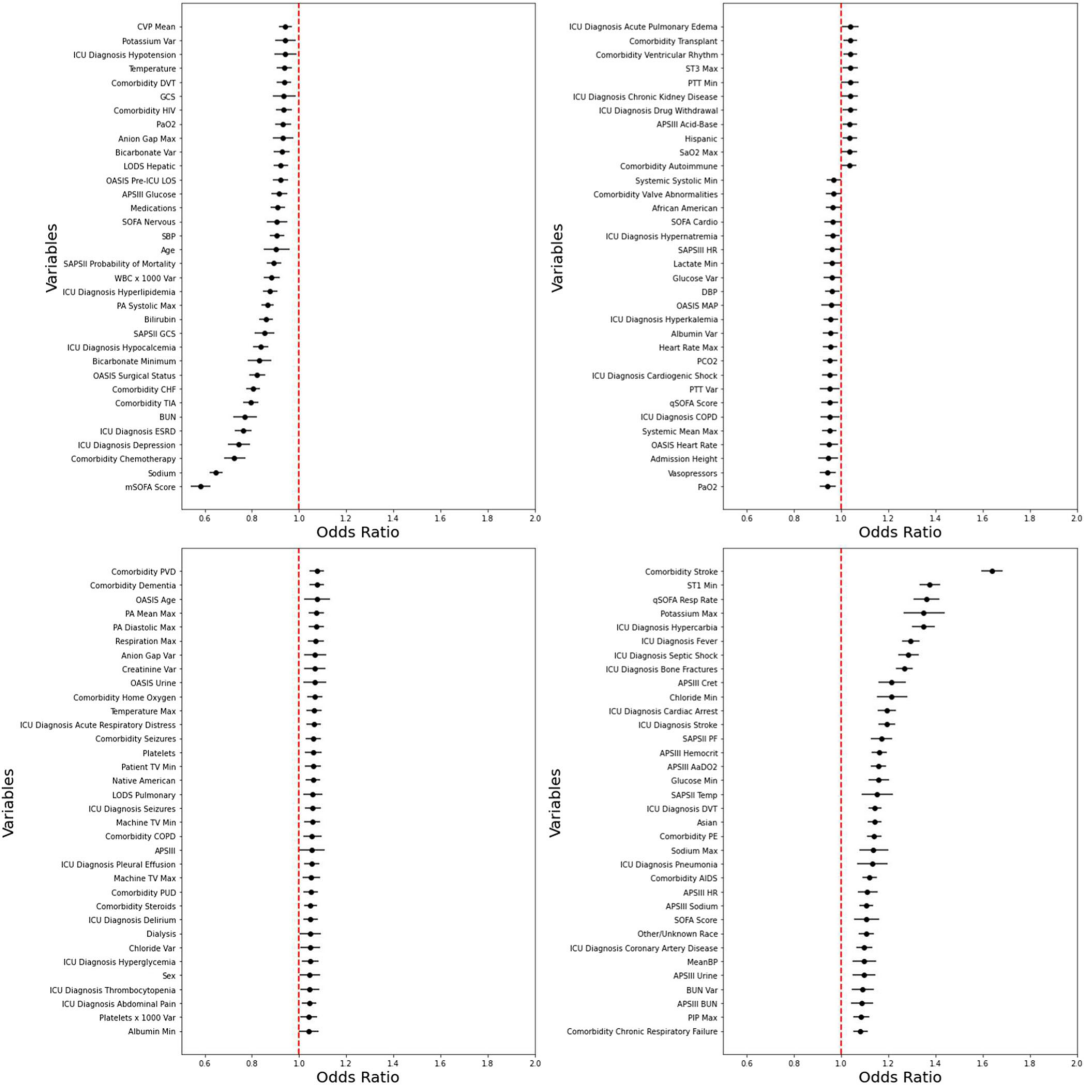
Statistically significant odds ratios from the logistic regression (LR) model. AaDO_2_, alveolar‐arterial oxygen gradient; BUN, blood urea nitrogen; CHF, congestive heart failure; COPD, chronic obstructive pulmonary disease; CVP, central venous pressure; DBP, diastolic blood pressure; ESRD, end‐stage renal disease; GCS, Glasgow Coma Scale; LODS, Logistic Organ Dysfunction System; mSOFA, Modified Sequential Organ Failure Assessment; OASIS, Oxford Acute Severity of Illness Score Set; PCO_2_, partial pressure of carbon dioxide; PF, pulmonary function; PIP, peak inspiratory pressure; PTT, partial thromboplastin time; PVD, peripheral vascular disease; qSOFA, Quick Sequential Organ Failure Assessment; SAPSII, Simplified Acute Physiology Score II; SAPSIII, Simplified Acute Physiology Score III; SOFA, Sequential Organ Failure Assessment; TIA, Transient Ischemic Attack.

To account for missing data, forward and multiple imputation by chained equations were used. For the remaining missing data, multiple imputations by chained equations were used. Descriptive statistics were presented as the median and interquartile range (IQR) for continuous variables and compared between both groups using the Mann‐Whitney *U* test. Categorical data were represented as frequencies and percentages and compared via a Chi‐square test. A p‐value less than 0.05 was deemed to be statistically significant. Furthermore, using logistic regression, odds‐ratios for tracheostomy placement were generated based on the DOA variables (Figure 2). All statistical analysis and modeling were performed via Python version 3.8 (Python Software Foundation) using the following packages: Pandas, SkLearn, and XGBoost.

## Results

### Patient Characteristics

The eICU‐CRD database contains 200,859 ICU admissions of 139,367 unique patients.^26^ Out of the entire cohort, 38,508 underwent IMV when admitted to the ICU, and 1605 patients received a tracheostomy. Further filtering the cohort for ICU stays greater than 48 hours and for adult patients, 36,253 patients remained.

A total of 347 features were extracted from the eICU‐CRD dataset. The baseline characteristics of the initial cohort (n = 35,034) are shown in Table 4. Table 4 describes the baseline characteristics amongst the “Tracheostomy” and “No Tracheostomy” groups following categorization for the binary classification model. The “Tracheostomy” group had more comorbidities such as atrial fibrillation, chronic kidney disease, chronic respiratory failure, congestive heart failure, and thyroid dysfunction. Additionally, this group exhibited significantly lower albumin, creatinine, lactate, potassium, PTT, and PT‐INR on the day of ICU admission. Furthermore, the tracheostomy group had higher ventilation parameters on the DOA such as PEEP, PIP, and total RR. Applying traditional multivariate analysis to all DOA variables, logistic regression odds ratio demonstrated 136 variables with statistically significant odds ratios of which 79 features had a positive association with the likelihood of tracheostomy and 57 features with a negative association with the likelihood of tracheostomy. Patients with a low modified SOFA (mSOFA) score, serum sodium, a comorbidity of chemotherapy, ICU diagnosis of depression, or ICU diagnosis of end‐stage renal disease (ESRD) were significantly less likely to experience a tracheostomy. However, patients with a comorbid stroke, minimum ST segment level of lead 1, quick SOFA (qSOFA) respiratory rate, maximum serum potassium, or ICU diagnosis of hypercarbia were more likely to undergo a tracheostomy.

**Table 4:** Baseline Characteristics Between the “Tracheostomy” and “No Tracheostomy” Cohorts

	Tracheostomy	No Tracheostomy	*P* value
Patient demographics	1449	33,585	
Age (years)	62.0 (52.0, 72.0)	65.0 (54.0, 75.0)	<.001
Male (gender)	875 (60.4%)	19,061 (56.8%)	.01
Female (gender)	574 (39.6%)	14,524 (43.2%)	.01
Weight (kg)	80.0 (65.0, 99.8)	81.6 (67.8, 98.8)	.16
Height (cm)	170.2 (162.6, 177.8)	170.2 (162.6, 177.8)	.2
Ethnicity			
White	1065 (73.5%)	26,105 (77.7%)	<.001
Black	186 (12.8%)	3563 (10.6%)	.01
Hispanic	77 (5.3%)	1347 (4.0%)	.02
Asian	36 (2.5%)	529 (1.6%)	.01
Other	72 (5.0%)	1752 (5.2%)	.72
PMHx			
Acute Renal Failure	446 (30.8%)	7161 (21.3%)	<.001
Acute Respiratory Distress Syndrome	713 (49.2%)	12,079 (36.0%)	<.001
Atrial Fibrillation	179 (12.4%)	3020 (9.0%)	<.001
Cardiac Arrest	117 (8.1%)	2374 (7.1%)	.16
Chronic Kidney Disease	137 (9.5%)	2366 (7.0%)	<.001
Chronic Respiratory Failure	157 (10.8%)	796 (2.4%)	<.001
Congestive Heart Failure	384 (26.5%)	6954 (20.7%)	<.001
Diabetes Mellitus	177 (12.2%)	4380 (13.0%)	.38
Hypertension	290 (20.0%)	5839 (17.4%)	.01
Intracranial Injury	807 (55.7%)	986 (2.9%)	<.001
Pleural Effusion	129 (8.9%)	1740 (5.2%)	<.001
Renal Failure	123 (8.5%)	2828 (8.4%)	.97
Seizures	104 (7.2%)	1962 (5.8%)	.04
Sepsis	885 (61.1%)	15,476 (46.1%)	<.001
Stroke	144 (9.9%)	2816 (8.4%)	.04
Thyroid Dysfunction	162 (11.2%)	2960 (8.8%)	<.001
Urinary Tract Infection	111 (7.7%)	1628 (4.8%)	<.001
Severity of illness			
LODS	4.0 (2.0, 7.0)	5.0 (3.0, 8.0)	<.001
SAPSII	64.0 (57.0, 72.0)	66.0 (58.0, 76.0)	<.001
SOFA	3.0 (1.0, 5.0)	3.0 (2.0, 6.0)	<.001
APS‐III	53.0 (36.0, 73.0)	53.0 (38.0, 75.0)	.07
SAPS‐III	64.0 (57.0, 72.0)	66.0 (58.0, 76.0)	<.001
APACHEIV	65.0 (47.0, 83.0)	66.0 (48.0, 87.0)	<.001
OASIS	37.0 (32.0, 44.0)	37.0 (31.0, 44.0)	.52
Laboratory values mean			
Albumin	2.9 (2.4, 3.4)	3.05 (2.5, 3.5)	<.001
Bicarbonate	25.0 (22.5, 28.5)	24.0 (21.0, 26.3)	<.001
Total bilirubin	0.6 (0.4, 0.9)	0.6 (0.4, 1.0)	.21
Creatinine	0.915 (0.7, 1.4)	1.025 (0.8, 1.6)	<.001
Hematocrit	33.2 (28.6, 38.3)	33.65 (29.1, 38.4)	.08
Hemoglobin	10.9 (9.2, 12.7)	11.1 (9.5, 12.7)	<.001
Lactate	1.7 (1.2, 2.8)	1.9 (1.2, 3.2)	<.001
Platelets ×1000	208.583 (159.2, 280.4)	193.0 (143.5, 252.0)	<.001
Potassium	4.0 (3.7, 4.4)	4.078 (3.8, 4.4)	<.001
PTT	31.0 (27.3, 37.0)	32.0 (28.0, 38.7)	<.001
PT–INR	1.175 (1.1, 1.4)	1.2 (1.1, 1.4)	<.001
paO_2_	122.0 (77.0, 188.0)	122.0 (81.0, 187.0)	.45
pcO_2_	42.0 (34.0, 54.0)	41.0 (33.0, 50.0)	<.001
Sodium	138.944 (136.0, 142.0)	139.0 (136.2, 141.4)	.36
BUN	19.0 (12.5, 31.0)	19.25 (13.2, 31.0)	.2
WBC x 1000	11.6 (8.8, 15.2)	11.617 (8.7, 15.5)	.79
Ventilator Settings Mean	2.9 (2.4, 3.4)	3.05 (2.5, 3.5)	<.001
PEEP	5.0 (5.0, 6.6)	5.0 (5.0, 5.6)	<.001
PEEP Max	5.0 (5.0, 8.0)	5.0 (5.0, 7.0)	<.001
PIP	23.31 (18.0, 28.3)	21.66 (18.0, 26.0)	<.001
PIP Max	28.0 (22.0, 34.7)	26.0 (21.0, 32.0)	<.001
Total RR	19.17 (16.0, 22.6)	18.0 (15.6, 21.5)	<.001
Total RR Max	24.0 (19.0, 29.0)	22.0 (18.0, 27.0)	<.001
VT, Set	491.62 (431.6, 560.6)	505.18 (449.9, 565.9)	.01
VT, Spont	495.55 (431.9, 558.9)	499.12 (414.5, 589.0)	.57
ICU admission diagnosis			
Acute renal failure	446.0 (30.8%)	7161.0 (21.3%)	<.001
Acute respiratory distress	160.0 (11.0%)	2441.0 (7.3%)	<.001
Acute respiratory failure	807.0 (55.7%)	13,946.0 (41.5%)	<.001
Acute respiratory distress syndrome	713.0 (49.2%)	12,079.0 (36.0%)	<.001
Atrial fibrillation	179.0 (12.4%)	3020.0 (9.0%)	<.001
Bacteremia	253.0 (17.5%)	3758.0 (11.2%)	<.001
Cardiac arrest	117.0 (8.1%)	2374.0 (7.1%)	.16
Chronic kidney disease	137.0 (9.5%)	2366.0 (7.0%)	<.001
Congestive heart failure	384.0 (26.5%)	6954.0 (20.7%)	<.001
COPD	269 (18.6%)	5871 (17.5%)	.3
Encephalopathy	820.0 (56.6%)	14,419.0 (42.9%)	<.001
Hypoxemia	154.0 (10.6%)	3458.0 (10.3%)	.72
Pleural effusion	129.0 (8.9%)	1740.0 (5.2%)	<.001
Pneumonia	861.0 (59.4%)	14,332.0 (42.7%)	<.001
Sepsis	885.0 (61.1%)	15,476.0 (46.1%)	<.001
Stroke	189.0 (13.0%)	2388.0 (7.1%)	<.001
Urinary tract infection	111.0 (7.7%)	1628.0 (4.8%)	<.001

Abbreviations: APACHEIV, acute physiology and chronic health evaluation IV; APS‐III, Acute Physiology Score III; BUN, blood urea nitrogen; COPD, chronic obstructive pulmonary disease; LODS, Logistic Organ Dysfunction System; OASIS, Oxford Acute Severity of Illness Score; paO_2_, partial pressure of arterial oxygen; PCO_2_, partial pressure of carbon dioxide; PEEP, positive end‐expiratory pressure; PIP, peak inspiratory pressure; PT, prothrombin time; PT–INR, prothrombin time–international normalized ratio; PTT, partial thromboplastin time; SAPS‐II, Simplified Acute Physiology Score II; SAPSII, Simplified Acute Physiology Score II; SOFA, Sequential Organ Failure Assessment; WBC x 1000, white blood cell count times 1000.

### Model Performance

**Figure 3:**
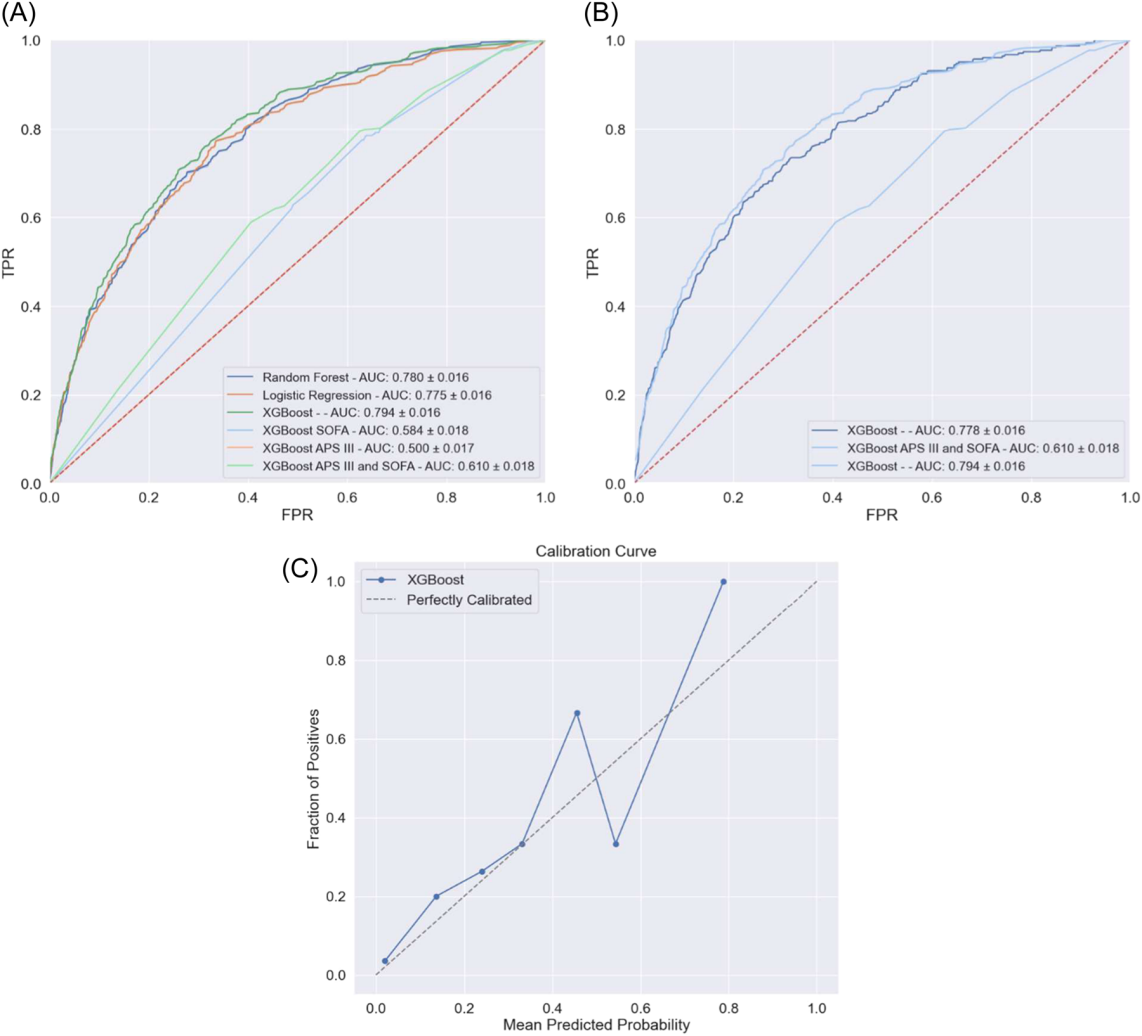
Performance metrics of the developed models (XGBoost, RF, and LR) compared with models using disease severity scoring systems variables.

The performance metrics of the different ML models used for predicting tracheostomy at ICU admission are shown in Figure 3 and Table 5. The XGBoost, RF, and LR prediction models trained with 331 DOA variables demonstrated similar fair performances. Specifically, the XGBoost model achieved an AUC‐ROC of 0.794 ± 0.016. The RF model achieved an AUC‐ROC of 0.780 ± 0.016, and the LR model achieved an AUC‐ROC of 0.775 ± 0.016. On the other hand, the XGBoost APS‐III and SOFA model performed marginally better than a random classifier with an AUC‐ROC of 0.610 ± 0.018. (Figure 3A). However, using 331 clinical features in the clinical environment would prove challenging for a physician; therefore, we aimed to retrain the XGBoost model on the 20 most important features found through recursive feature pruning to create an “XGBoost Pruned Model.” This allows clinicians to realistically apply the model while removing extraneous variables that may negatively bias the model. After pruning the DOA variables to only 20 predictive variables, the XGBoost Pruned model exhibited minimal reduction in its performance. The AUC‐ROC had a minimal reduction in its performance from 0.794 ± 0.016 to 0.778 ± 0.016 (Figure 3B).

**Table 5:** Performance Metrics of Models Predicting “Tracheostomy Need” Using Day of Admission Variables

	RF DOA	LR DOA	XGBoost DOA	XGBoost DOA Pruned	XGBoost SOFA	XGBoost APS‐III	XGBoost SOFA + APS‐III
AUC ROC	0.780	0.775	0.796	0.778	0.584	0.500	0.610
Mean precision	0.083	0.087	0.087	0.115	0.098	0.521	0.237
Sensitivity	0	0.294	0.5	0	0	0	0
Specificity	0.958	0.959	0.959	0.957	0.957	0.958	0.959
Positive predictive value	0	0.014	0.004	0	0	0	0
Negative predictive value	1	0.999	1	1	1	1	1
False positive rate	0.042	0.041	0.041	0.043	0.043	0.042	0.041
False negative rate	0	0.706	0.5	0	0	0	0
False discovery rate	1	0.986	0.996	1	1	1	1
*F*‐Score	0	0.026	0.008	0	0	0	0
Accuracy	0.958	0.958	0.959	0.957	0.957	0.958	0.959

The pruned XGBoost DOA model includes 20 features determined through recursive feature elimination (RFE).

The calibration curve for the XGBoost DOA prediction model is depicted in Figure 3C. The model exhibited a low Brier score of 0.0390, indicating acceptable calibration. A Brier score of 0.0 represents a model with perfect skill, while a score of 1.0 indicates a model with no skill.

### Feature Importance Analysis and Explainability

**Figure 4:**
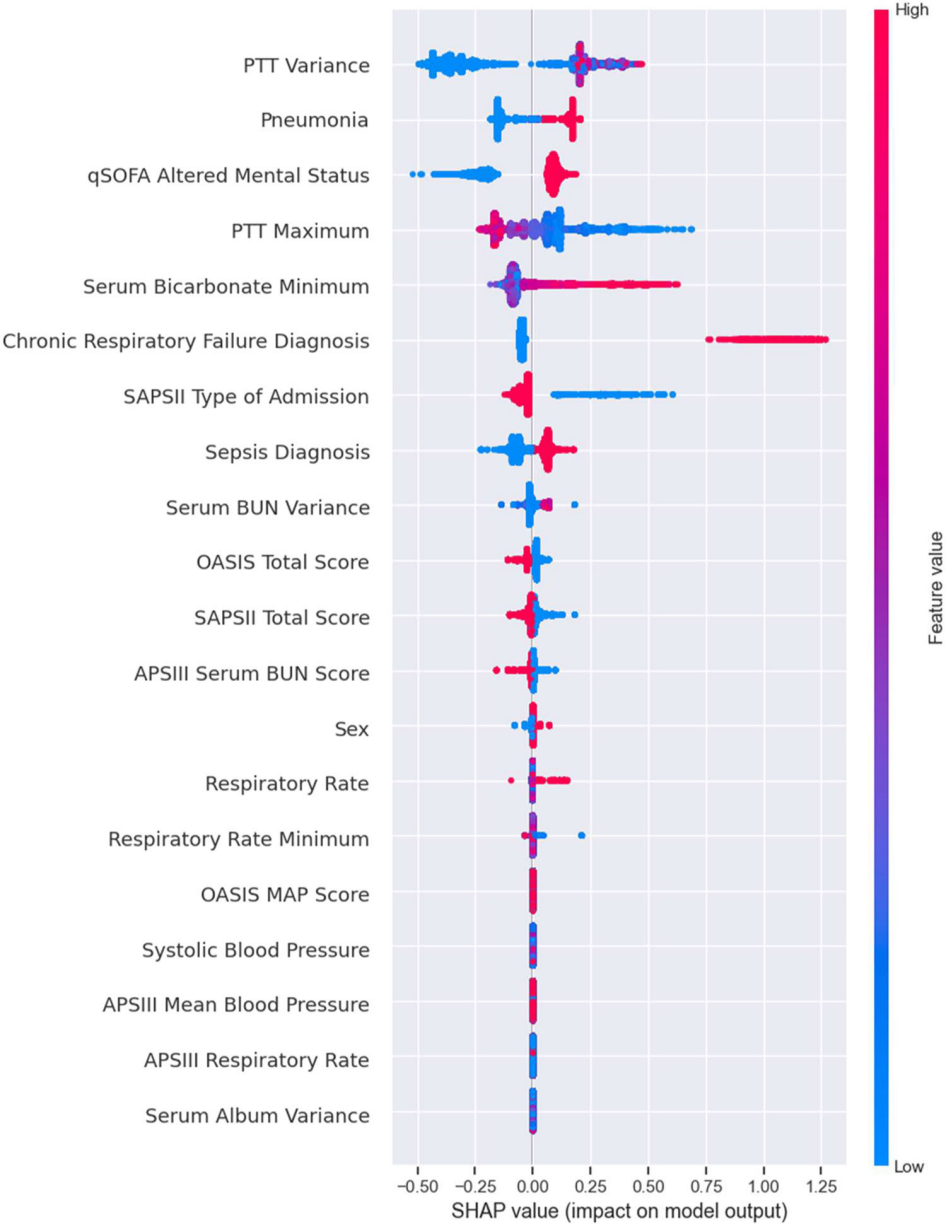
The contribution of each feature towards the output of the “pruned” model using the SHapley Additive exPlanations (SHAP) values for the prediction of tracheostomy need. APSIII, Acute Physiology Score III; BUN, Blood Urea Nitrogen, Oxford Acute Severity of Illness Score, PTT, partial thromboplastin time, qSOFA, Quick Sequential Organ Failure Assessment; SAPSII, Simplified Acute Physiology Score II.

The results of The SHAP analysis for the XGBoost Pruned prediction model are depicted in Figure 4. The analysis highlighted the significance of certain features in predicting tracheostomy. Notable variables with high feature importance included PTT variance, congestive heart failure, admission with pneumonia, and comorbidity of chronic respiratory failure.

## Discussion

### Main Findings

In this study, our objective was to develop a ML model for predicting the need for tracheostomy in patients admitted to the intensive care unit who were undergoing mechanical ventilation. We aimed to identify factors that could predict the need for this surgery from the onset of admission, allowing for earlier intervention, less resource utilization, and ultimately reduced intubation‐related complications.

Our study demonstrated that a prediction model based on clinical variables obtained on the DOA to the ICU and after the first 24 hours of mechanical ventilation can accurately predict the need for a tracheostomy. The performance metrics demonstrate that the XGBoost model is fairly accurate and reliable in its predictions with an AUC‐ROC of 0.794. This suggests that the model has a fair ability to distinguish between patients who will undergo a tracheostomy or not and with a specificity of 0.959, our model can reliably identify patients suited for tracheostomy.

In our analysis, we initially extracted 331 variables from the database. However, through data pruning using RFE techniques, we selected the top 20 features for further evaluation to reduce the data collection burden for clinicians and make it clinically applicable. We then compared the performance of 2 models: the XGBoost model with 331 features and the pruned XGBoost model with 20 features. The results showed that both models performed similarly: the XGBoost achieved an AUC‐ROCs of 0.796, while the pruned XGBoost model with only 20 features attained an AUC‐ROC of 0.778, demonstrating the reduction of features minimally reduced model performance. These findings indicate that the 20 selected features were highly informative and captured the key information necessary for accurate predictions. This highlights the potential for simplifying the model without significant loss in performance, which can enhance efficiency, interpretability, and usability by clinicians.

Often ML algorithms present the “black box problem” where the models are not easily interpretable by humans, which can impair trust in these models.^33^ Therefore, we conducted an explainability assessment using Shapley values. This method not only determines the importance of each feature to the model's performance but also quantifies directly the impact of each input on the model's output. After conducting an explainability assessment, we found that the presence of an admission diagnosis of pneumonia or sepsis and a comorbidity of chronic respiratory failure were associated with tracheostomy. Casamento et al through a systematic review of 12 observational studies of critically ill trauma patients demonstrated that pneumonia was a significant predictor of tracheostomy.^34^ In a prospective observational study of patients with severe stroke, Schneider et al found the absence of sepsis was associated with a decreased need for tracheostomy.^35^ XGBoost being a nonlinear machine learning algorithm also found the absence of sepsis as a negative predictor of tracheostomy need (Figure 4). Parreco et al also found similar results when developing their tracheostomy predictor on the Multiparameter Intelligent Monitoring in Intensive Care III (MIMIC‐III) dataset as their model relied on the LODS pulmonary score as the primary predictor for tracheostomy, encapsulating the respiratory status of a patient with chronic respiratory failure.^36^ Windsor et al in a study of tracheostomy predictors in the NICU of 73 preterm patients found those with severe pulmonary disease were more likely to undergo a tracheostomy.^37^ Additionally, several daily laboratory values on the DOA:PTT variance, PTT maximum, and minimum serum bicarbonate were also predictive factors of tracheostomy need. Clinicians use the PTT to estimate the intrinsic pathway of the coagulation cascade, measuring an individual's coagulation ability. This confounds the relationship found in this study between the PTT and tracheostomy risk because clinicians will avoid the intervention when the patient is at an increased risk for bleeding. Clark et al developed the model I‐Trach to predict tracheostomies for PMV and found that serum bicarbonate <20 mEq/L correlated with a tracheostomy need.^38^

Comparing the features deemed important by the pruned XGBoost model with the logistic regression odds ratios, there appears to be agreement between the 2 models that a comorbidity of chronic respiratory failure, ICU diagnosis of pneumonia, or ICU diagnosis of septic shock led to a greater likelihood of tracheostomy placement for mechanically ventilated patients. However, the logistic regression model demonstrated statistically significant variables including those with a comorbid stroke, minimum ST segment level of lead 1, quick SOFA (qSOFA) respiratory rate, maximum serum potassium, or ICU diagnosis of hypercarbia. The pruned XGBoost model failed to identify these features as important to predicting tracheostomy need. Logistic regression models assume simple linear interactions between clinical features to estimate the likelihood of an outcome whereas XGBoost models assume a nonlinear relationship between the independent variables to estimate an outcome. This assumption of a nonlinear relationships allows XGBoost to better capture higher orders of complexity between features of the dataset to produce a more accurate prediction. Furthermore, the XGBoost DOA model outperformed the LR DOA model in classifying patients, suggesting greater predictive power in the features that XGBoost model deemed significant.

Considering the heterogeneity of patient presentations and the contribution of numerous clinical variables, machine learning enables precision care medicine. The objective of the predictive model in this study was to develop a clinical decision support tool that considers a combination of patient variables to predict tracheostomy need—a clinical problem that traditional predictors alone have struggled to reliability address within the clinical setting.^23^ SOFA and APSII have traditionally been used as predictors of PMV, which can serve as a proxy for tracheostomy risk.^39^ The XGBoost model trained solely on APSIII and SOFA performed as well as a random classifier with no ability to reliably predict tracheostomy need; whereas both our pruned and full XGBoost models performed fairly reliably as discriminators of tracheostomy need.

Although prolonged MV patients constitute a small percentage of all MV patients, they disproportionally consume significant ICU resources.^6^ More importantly, PMV patients are at risk for developing ventilator‐associated infections, ventilator‐associated lung injury, needing prolonged sedation, physical deconditioning, and long‐term cognitive and psychological sequelae such as depression, anxiety, and PTSD.^40^, ^41^, ^42^ In the setting of PMV, patients who received tracheostomies experienced lower in‐hospital mortality, greater weaning rates, and shorter ICU length of stays.^15^, ^16^ By accurately predicting tracheostomy risk, our model can potentially aid in reducing PMV‐associated complications.

Placing a tracheostomy is not without risks. Possible complications include pneumothorax, bleeding, subglottic stenosis, tracheoesophageal fistula, vocal cord dysfunction, stomal granulation, persistent tracheal fistula, and scarring.^10^ Therefore, effectively ruling out patients unfit for a tracheostomy would reduce unnecessary tracheostomy‐complications. Young et al noted in the TracMan Trial comparing the mortality benefits of early vs late tracheostomy in adult patients on mechanical ventilation that approximately 40% of early tracheostomies were unnecessary.^43^ This suggests that a significant number of patients may be unnecessarily exposed to potential tracheostomy‐associated complications. The pruned XGBoost model trained on DOA variables had a specificity of 0.957, suggesting the model can effectively eliminate patients inappropriate for a tracheostomy and may reduce unnecessary tracheostomies (Table 5).

Big data has enabled machine learning to offer many benefits over conventional statistical approaches by revealing complex associations among heterogeneous data and providing predictions for diagnosis or prognosis.^24^ However, it is essential to develop the model in a systematic way to ensure reliable predictions for users.^24^, ^44^ The predictive models created in this study adhered to the TRIPOD guidelines, providing full transparency into the model's development and further validity.^25^ Previous studies on tracheostomy need prediction have shown fair performance metrics, but they fail to quantify the extent to which each feature contributes to the model's prediction.^36^ In contrast, we used the SHAP explainability method to select the top 20 features and illustrated the input's effect on the model's output.

### Strengths

The eICU‐CRD database contains a vast amount of data for over 139,367 patients from 208 hospitals located throughout the United States, which allows for a comprehensive examination of multiple factors associated with tracheostomy placement for PMV. It also contains detailed clinical data which enables the ability to assess a wide range of variables that could contribute to tracheostomy need and contains real‐time patient data reflecting the complexities and challenges encountered in a real‐world clinical setting. Other papers have relied solely on severity of illness scores or laboratory and vital values from the DOA, but our model combined data from demographic, clinical, severity of illness scores, laboratory, daily vitals, and mechanical ventilation settings to predict tracheostomy need.^36^, ^38^ As such, our model used the most comprehensive set of information to select the top 20 features that strongly drive the need for this multifactorial clinical question.

### Limitations

Several limitations afflict this study. First, this is a retrospective study, and therefore, causality cannot be established. This is valuable in generating hypotheses; however, prospective data would provide robustly founded conclusions. Therefore, prospective studies must be performed to validate our models' conclusions. Second, the data captures only 2 years of clinical data from 2014 to 2015, preventing longitudinal analysis of patient trajectories. Third, while we identified several clinical features that are predictive of tracheostomy, there may be other factors that were not captured in our analysis that could also be important predictors. Future studies should consider including additional variables to improve the accuracy of the prediction model. The fourth limitation is the binary classification model only predicts whether a patient is at risk of tracheostomy. However, because there is a significant variance in when institutions perform tracheostomies in PMV patients, a tool that predicts when to perform a tracheostomy in this population would be more valuable.^13^, ^20^ Future models will likely include time‐series models, which provide a live prediction of tracheostomy need. Lastly, there was a significant class imbalance present within the dataset where there are 23 patients who did not experience tracheostomy to every 1 patient who did (Table 5).

## Conclusions

We have developed a machine‐learning model for predicting the risk of tracheostomy in mechanically ventilated patients using a large multicenter clinical database. Our model achieved good discrimination and can be used in clinical practice to help identify patients at high risk of tracheostomy at the onset of ICU admission. Future prospective studies are needed to validate our findings and assess the clinical utility of the prediction model.

## Author Contributions

**Matthew Nguyen**, data collection and analysis, manuscript preparation and final manuscript revisions; **Ameen Amanian**, project inception and revisions of the final manuscript; **Meihan Wei**, data collection and analysis; **Eitan Prisman**, project inception, preparation of research protocol, and manuscript editing critical for important intellectual content; **Pedro Alejandro Mendez‐Tellez**, project inception, preparation of research protocol, and manuscript editing critical for important intellectual content.

## Disclosure

### Competing interests

None.

### Funding source

None.
